# Genes, Gender, Hormones, and Doping in Sport: A Convoluted Tale

**DOI:** 10.3389/fendo.2017.00251

**Published:** 2017-10-12

**Authors:** Alan D. Rogol, Lindsay Parks Pieper

**Affiliations:** ^1^Department of Pediatrics, University of Virginia School of Medicine, Charlottesville, VA, United States; ^2^Department of Sport Management, Lynchburg College, Lynchburg, VA, United States

**Keywords:** gender, genes, hormones, athletic performance, therapeutic use exemption

## Abstract

We are writing this piece in the aftermath of the 2016 Olympic Games in Rio de Janeiro, Brazil. Each of the words in the title plays a role(s) in deciding who may compete, especially who may compete as a woman. We shall be careful to disentangle the issues of genes and gender from hormonal levels of the potent androgen testosterone, and very clearly demarcate these *natural* occurrences from those of doping, for which the World Anti-Doping Agency has established strict guidelines. These elements became conflated in the aftermath of the Court of Arbitration of Sport’s decision, now more than 2 years ago, concerning the teenage Indian sprinter, Dutee Chand. Although many people associate hyperandrogenism with doping and gender determination, each is different and has a distinct function.

## History of Sex Testing in Female Athletes

Sex verification and anti-doping measures are not new in sport. Because many western practitioners viewed women as too weak for strenuous activities, sport developed as a male-dominated activity. Therefore, when female athletes started to participate—and excel—in sport, fears of masculinization and male imposters surfaced (1).

To combat the threat of men masquerading as women, the International Association of Athletics Federations (IAAF) and International Olympic Committee (IOC) sporadically checked female athletes’ anatomies before competition. The most well-known example occurred in the 1936 Berlin Olympics, when officials forced US runner Helen Stephens to undergo a sex test (2). Her record-setting time in the 100-m race concerned the sport authorities. After the Berlin Games, World War II stopped both sport and random testing.

When elite competitions resumed, the IAAF required a physician’s letter for all female track and field athletes that indicated the athlete’s sex. The IOC did the same 2 years later, in 1948 (3). Worried that unscrupulous individuals might forge documents, the IAAF instituted a compulsory anatomical check in the 1966 British Empire and Commonwealth Games. Although sport officials considered the “naked parades” successful in deterring male imposters, the athletes’ dislike of the procedure pushed the IAAF to introduce a buccal smear test in 1967. This test, also known as the Barr body test, assessed chromosomal composition with the Barr body representing the second (inactive) X chromosome (2). The IAAF instituted a Barr body test for all female athletes at the 1967 European Cup Track and Field Event.

The IOC instituted a buccal smear test in 1968. At the 1968 Grenoble Winter Olympics, 20% of female athletes underwent the exam on a trial basis. Believing the method successful in eliminating male masqueraders, the IOC extended the policy to all women at the 1968 Mexico City Summer Olympics. The IAAF and IOC justified the policy as a means to unearth men disguised as women; yet, neither organization ever discovered an imposter. Instead, the verification measures prohibited women with certain differences of sex development (DSD). Women with androgen insensitivity syndrome most frequently faced disqualification. The number of prohibited athletes remains unknown as the IAAF and IOC kept the information confidential but estimates range from one-to-two in each Olympics to one-out-of-every four hundred competitors. For example, in the 1985 World University Games, Maria José Martínez Patiño was disqualified for “failing” the sex test. She wrote a harrowing account of the public scrutiny she experienced as a result (4).

Because testing disqualified some women with DSD, members of the medical community protested the policy from its onset. Endocrinologists and geneticists pointed out that no one measure could unequivocally identify sex (5). When protests increased, the IAAF abandoned the practice in 1992. The IOC responded by introducing a new iteration of verification: polymerase chain reaction testing. PCR tests assessed the SRY (testis-determining gene) to identify (male) sex (6). From 1992 to 1999, the IOC employed this practice. The medical community continued to oppose the policy, pointing out the problems with it in sex determination and the likelihood for false positives. The IOC’s Athletes’ Commission also joined in the disapproval of testing, convincing the IOC to terminate the practice in 1999.

Compulsory gender verification stopped with the IOC’s decision; however, the IAAF and IOC still allowed for suspicion-based testing. In 2011, the IAAF and IOC reintroduced gender policies, this time targeting women with hyperandrogenism. According to sport officials, women with hyperandrogenism possessed an unfair advantage for naturally producing higher levels of testosterone (up to those of the lower limit of normal for adult men) than other women. The IAAF and IOC therefore instituted 10 nmol/L of testosterone as the cutoff for women’s competition. Chand protested the IAAF policy in 2014 and the Court of Arbitration of Sport (CAS) suspended it the following year, citing the need for scientific verification of the advantages afforded by increased testosterone levels.

While waiting for a final decision from CAS, scholars and scientists from various backgrounds have put forward different schemas to include women with DSDs into elite sport. Citing the importance of human rights and the historic mistreatment of women with DSDs, some recommend reducing biological considerations from eligibility. For example, Drs. Myron Genel, Joe Leigh Simpson, and Albert de la Chapelle suggest athletes born with DSDs and raised female be allowed to compete (7). By contrast, others support the IAAF’s and IOC’s 10 nmol/L threshold. Dr. David Allen, for example, suggests the cutoff is a pragmatic necessity to preserve fairness for women without DSDs (8).

We argue that women with hyperandrogenism possess a natural edge not dissimilar to those afforded by certain genetic makeups and other hormonal conditions. We thus recommend that individuals born and raised through puberty as female and who continue to affirm the female gender compete in the women’s category, thereby obviating testosterone testing. Thoughts on and suggestions for transgender participation fall outside the scope of this study.

## Genes

Let us turn to genes. Are there genetic factors that permit enhanced athletic performance? Of course there are, from the almost trivial—if you want to be an Olympic champion, then you better pick your parents (actually probably several generations back) very carefully! Beyond these multiple genes for growth, muscle function, and cardiopulmonary function, there are certain individual genes that clearly affect athletic performance and permit exceptional function.

From the past, athletes such as Wilt Chamberlain (7′ 2″, 2.18 m) performed exceptionally as a basketball player and Willie Shoemaker (4′ 11″, 1.5 m; 110 lb, 50 kg) as a horse-racing jockey. Their genetic makeup was quite different, but at the extremes of the physiological range for height. These differences were undoubtedly due to the individually small contribution of hundreds of genes (9). They trained exceptionally hard and were permitted to compete at the highest levels. For these and many other athletes, it is likely that these genetic variations had a major role in their selection of sport.

What about single genes? There are many examples that offer an athletic advantage, but we shall choose just a few that note the span of effect. Examples include the erythropoietin receptor (EPOR), the androgen receptor (AR) [complete androgen insensitivity (CAIS)], and some hormonal excesses and deficiencies (virilizing adrenal hyperplasia and genetic growth hormone deficiency, and likely myostatin deficiency).

Eero Mantyranta was a Finnish long distance skier with multiple Olympic gold medals. Not only did he win a series of gold medals but he also finished minutes ahead of his nearest competitors in long distance events—unheard of time differentials compared with previous competitions. How did he do it—by assiduously training and the right mind-set—but also because his hemoglobin concentration is up to 65% higher than the average male, but without the significant problems of rheology that have occurred in some cyclists who abused erythropoietin or were infused with autologous blood. One might immediately consider blood doping, a World Anti-Doping Agency (WADA)-prohibited method or receiving erythropoietin; however, it was shown that Mantyranta and his family have a single base-pair mutation in the EPOR gene that permits his bone marrow to continue to manufacture erythrocyte precursors that mature to oxygen-carrying red cells (10). de la Chapelle had spent a large part of his scientific career tracking genes among the Finnish and solved the finding of the high hemoglobin levels in the early 1990s by discovering the single mutation in the EPOR (11, 12). Should this genetic mutation disallow Mantyranta from competition? We do not think so.

A second gene is myostatin, a protein that places a brake on the size of muscle. Homozygous deletion of this gene is well described in Belgian Blue cattle, which are excessively well muscled (double muscled) with a decrease in body fat (13). The human homolog of this gene is well known with a single child with a mutation in this gene reported (14). He was well muscled at birth and has continued to grow (at least up to 4 years of age) as a child “Hercules” with commensurate strength. The gene mutation in the myostatin gene with very low myostatin levels is homozygous and accountable for the phenotype. It is too early to define athletic performance in this boy. Intriguingly, his heterozygous mother (and heterozygous whippet-dogs—bred unknowingly for speed) was a professional sprinter (15).

Another condition is that of familial partial lipodystrophy, Dunnigan variety. Most women have fat loss from the extremities, abdomen, and thorax with excessive subcutaneous fat in the facial and supraclavicular areas. However, it is also associated with increased muscularity that occurs with the loss of subcutaneous fat (16). Many may have insulin resistance, hirsutism, and menstrual abnormalities. There are several genetic varieties that likely affect the LMNA gene, which encode nuclear laminins.

A fourth gene is that for the AR. Those without any function at all of this gene appear at birth as normal females, but only have the outer one-third of a “vagina” given that it is derived from non-Mullerian structures (see below). The rest of the female reproductive tract is missing, since the 46,XY females have testes that had produced anti-Mullerian hormone responsible for the disappearance of the uterus, Fallopian tubes, and the inner two-third of the vagina.

Young adolescents with this condition appear quite normally female with breast development but have no pubic hair and are primarily amenorrheic. Although the testosterone levels are often well above the upper limit of normal for adult males, there are no testosterone effects. On the contrary, this reservoir of testosterone is a precursor for the aromatase enzyme and leads to significant estrogen (mainly estradiol) levels. The phenotype with regard to body habitus (gynecoid) and normal breast development is entirely female except for the reproductive system, including sexual hair. Under the previous testing paradigm to permit the athlete to compete as a woman, these women did not have a Barr body (second X chromosome), had the 46,XY karyotype and the SRY (testis-determining gene). Clearly those with CAIS have no athletic advantage from the very high T levels, although they are likely to be taller than the average 46,XX woman given some height-determining genes on the Y chromosome and perhaps some that increase lean body mass (17). Mutation of this gene is found in fewer than 1 in 20,000 in the general population but is relatively common in elite female athletes [noted as 1/421 (17) and 1/423 at the 1996 Atlanta Olympic Games (2)].

The more profound difficulties come forward in those with partial androgen insensitivity syndrome—a spectrum from the unquestionably female phenotype (as in CAIS) to virtually completely male—all have the 46,XY karyotype—as well as those with enzyme deficiencies, usually along the steroid hormone pathway to cortisol, some of which affect the ovary and testis as well. It is these later conditions that lead to high *endogenous* testosterone (and other androgen) levels and the meandering tale of who is permitted to compete as a female (see above for a more in depth history of the evolution of testing of who should be permitted to compete as a female). Even the last iteration, testosterone levels must be below those of the lower limit of the male. That standard is now held in abeyance awaiting data to demonstrate that it is these levels *per se* that “completely” explain the differences in athletic performance between men and women and a “sliding” scale that predicts better performance in those women with high testosterone levels and androgen sensitivity. As noted several times above, these issues should not be confused with doping with androgenic agents.

The 5-alpha reductase gene is another that can be mutated and lead to a disorder of sex development. Such 46,XY individuals will have low levels of dihydrotestosterone and may be so undervirilized that they are unambiguously raised as female. Four athletes from the developing world are presented in a report that noted their diagnostic procedures and concluded with gonadectomy so that they could compete. Issues of fairness, medical ethics, and complete informed consent were not resolved (18).

## Hormones

Let us move to the subject of hormones. Acromegaly is a condition caused by excess growth hormone as an adult, but gigantism as well if the hormonal excess began before the epiphyses of the long bones have closed. There have been several players in the National Basketball Association with this condition causing excessive tall stature—a plus for a basketball player despite the other less salutary effect of the condition. Men who have very high levels of endogenous testosterone, whether producing enhanced athletic performance or not, are permitted to compete in men’s sports.

Extreme short stature may be caused by simple growth hormone deficiency. The substance (hormone) missing is hGH and may be completely replaced by recombinant (r)hGH. However, this is a banned agent on the WADA list. Very similar issues would occur for boys or men without testicles and the use of testosterone, another banned substance.

This brings us to the concept of a therapeutic use exemption (TUE), explained in more detail below, to administer a prohibited medication, whose necessity is certified by a qualified medical specialist. Over the past several decades, WADA and other organizations have sought to harmonize the granting of a TUE across sport and countries leading to an “international standard” TUE (ISTUE). This is the entry point into the system for pediatric and internal medicine endocrinologists for they are the medical specialists who will prescribe the banned substances (likely mainly testosterone and rhGH) as therapeutic agents for their athlete patients. They will need to learn how to provide the proper information about the diagnosis, as well as the necessity for the banned substance notably given that there are no non-banned substances that can be substituted. The WADA and USADA websites have instructions as well as “forms” for the requested information (19).

Where the realities of genes, gender, and hormones collide is in 46,XX or 46,XY karyotype athletes who have androgen levels above the upper limit for women. There are a number of other conditions for which androgen levels are raised above the reference range for females. The largest groups are those with polycystic ovary syndrome, and the women may have slightly raised testosterone levels to those several times the upper limit of the reference range. Women athletes with PCOS are found in greater numbers than in the general population (18). No specific gene has been implicated, and the genetics are complex. Differences in sex development are also conditions in which androgen levels, specifically testosterone, are raised. Taken as a whole, these conditions are approximately 140-fold more common in the elite athlete population than the general population (20).

After a long history of testing, the latest iteration accepted by the IAAF and the IOC, which calls for levels of total testosterone to be below 10 nM, the lower physiological limit for a young adult male, was adopted in 2011 (21) and overturned by the Court of Arbitration in Sport in July 2015 (22). The decision included the caveat that a defined upper limit of testosterone concentration to compete as a woman would be acceptable if clear data were presented within 2 years of the decision. That was the importance of the Dutee Chand appeal of her banishment for an adverse analytical finding (that is, non-doping) to the CAS. The Caster Semenya case from 2010 had a reprise at the Rio Olympic Games as the South African athlete won the gold medal in the women’s 800-m run.

Why the controversy? Should the levels of testosterone (alone) be the arbiter, irrespective of other naturally occurring variations? There are two large sets of data from elite athletes (23, 24). The methods of sampling—when in comparison to trans-meridian travel, time from exercise, issues of birth control pills, and time within a natural menstrual cycle—differ. The findings of relatively small numbers of athletes above the female range also differ given that the latter excluded athletes who were found to have doped or had a DSD. As noted by the CAS in the Dutee Chand case, the Court was not swayed about any data in the literature that “excess” testosterone levels were the *sole* factor in female athlete performance when a woman with high testosterone levels competed against women with lower levels. They left a two year window for such proof, before the stay from the 10 nmol/L level became permanent.

Caster Semenya and Dutee Chand are at the tip of the spear to make a clear distinction between doping—illegal as drugs or methods (see below), and natural, albeit rare, genetic conditions that confer increased physiologic capacity or just the routine collection of multiple genes that act and interact to affect height, cardiopulmonary physiology, speed, and power that may make marginal differences among athletes. One must also note that physical and psychological issues of training and competition will permit these differences in the genome to manifest as athletic performance.

These physiologic differences are considered in the rather new concept of an athlete’s biological passport. This concept is most mature for hematologic parameters. The intraperson changes over time should be within a smaller range than for a population of the same gender and age. If one has a mutation that affects hemoglobin production, as in Mantyranta, then the levels ought to be very high all of the time. The issue here is that marked fluctuations may indicate doping. A similar module for androgens is being tested, and it is likely that there will soon be a new chapter in the athlete’s passport.

## Doping

Doping includes the use of an expedient (substance or method) that is potentially harmful to athletes’ health and/or capable of enhancing their performance, or the presence in the athletes’ body of a prohibited substance or evidence of the use thereof or evidence of the use of a prohibited method. Although sex testing and anti-doping measures historically served different purposes, many people conflated the two practices from their introduction. In 1967, the IOC introduced its first prohibited substance list. Banned substances included alcohol, amphetamines, cannabis, cocaine, ephedrine, opiates, and vasodilators. The IOC lacked the ability to test for anabolic steroids until 1975 (25). Because of the simultaneous introduction of the two tests, and the fact that the IOC Medical Commission oversaw both procedures, many people believed the practices served a singular purpose: to eliminate masculine women from competition. But genes, hormones, gender, and doping are not one and the same!

The World Anti-Doing Code (Code) has been instrumental in introducing the concept of “non-analytical” rule violations. Non-analytical rule violations have allowed anti-doping organizations to apply sanctions in cases where there is no positive doping sample, but where there may still be evidence that a doping violation has occurred (e.g., through a combination of three missed tests/whereabouts failures; longitudinal testing; evidence brought forward through an investigation).

The Code works in conjunction with five International Standards aimed at bringing harmonization among anti-doping organizations in various technical areas, namely:

- Prohibited list
- Testing and investigations
- Laboratories
- TUEs
- Protection of privacy and personal information

Thus, these issues are clearly separate and distinct from those of genes, gender, and hormonal levels and have been recently reviewed (26).

The TUE or as it is standardized across sports and countries, the international standard (ISTUE) is a critical area for pediatric and internal medicine endocrinologists to interact with the national and international sporting bodies. Its components include the following:

- The complete medical details including the history, clinical findings, and investigation must be submitted;
- The necessity to administer the prohibited medication including the dosage, route, and frequency of administration must be certified by a suitably qualified medical specialist;
- The medical necessity to administer the prohibited substance cannot be the result, wholly or partially, of prior use of a drug from the banned classes or banned methods.

Additional investigations requested by the Medical Advisory Committee (MAC) will be undertaken at the athlete’s or his/her National Olympic Committee’s expense. It should be noted that any doctor who provides the MAC with false information will be ineligible to be accredited as an Olympic team doctor or official. Finally, under *no circumstances* will permission be given to use any synthetic anabolic steroid. If an active androgen is necessary under the specific TUE, then that will be a form of testosterone. Virtually all TUEs, but especially those for endocrine active agents have conditions imposed for monitoring for efficacy and safety, especially to be sure that they are not taken in performance-enhancing amounts. Those conditions clearly indicate that the TUE is for the *replacement* of the missing hormone and not for performance enhancement.

## Epilogue

The participation in and the “rules” for who may compete in women’s sports traverses a long and winding historical pathway. Inextricably intertwined are the notions of femininity, the incorrect physiological context of what is too strenuous for a female athlete, the overarching concern that women’s sport would be overrun by male imposters, and tangentially doping, especially with androgens. Although genes, hormones, and doping have coalesced in conversations about female competitors, all have different functions and results.

Genes and hormones, along with environmental variables, play an instrumental role in who excels in elite sport. Various genetic and hormonal conditions provide athletes with certain biological tools that are advantageous in sport. Typically, these athletes are neither penalized nor removed from competition for possessing a natural edge; however, women born and raised as female with genetic conditions that naturally produce testosterone above the IAAF- and IOC-prescribed 10 nM are barred. Because their testosterone production is natural, and not a result of performance-enhancing substances, these individuals with genetic conditions that alter androgen levels should be considered no different from others with genetic conditions that create extremes in height, body proportions, oxygen consumption, muscle bulk, and morphology. In other words, the natural advantages found in some women parallel the competitive edges enjoyed by male athletes. We therefore argue that the 10 nM cutoff unfairly penalizes a biological benefit for individuals born and mature as female while other genetic and hormonal assets go unchecked.

Women’s sport is clearly quite different than it was just a few decades ago and very much different than a century ago. The recent Olympic Games in Rio de Janeiro showcased both men and women as athletes including events that only recently have crossed the sex barrier, such as the pole vault and wrestling.

Women can and do compete at the highest levels in many sports and demonstrate that with proper opportunity, training, and national and international support, they shall continue.

## Author Contributions

Both authors conceived the subject, partook in drafting the first version, and critiqued and wrote each succeeding draft.

## Conflict of Interest Statement

AR consults for AYTU BioScience Therapeutics and Acerus Pharma, both of which produce testosterone; AR also consults for Ammonett LLC, Versartis, and NovoNordisk, all of which produce products related to the growth hormone axis. LP declares that the research was conducted in the absence of any commercial or financial relationships that could be construed as a potential conflict of interest.
